# Artificial cell vesicle-mediated delivery of *Catharanthus roseus* (L.) G. Don-derived vinca alkaloids for enhanced antitumor efficacy

**DOI:** 10.3389/fbioe.2025.1703637

**Published:** 2025-10-15

**Authors:** Xiaodong Zhuang, Liangjiu Huang, Risheng Liu, Liwen Guan, Xingyue Fang, Ting Ma

**Affiliations:** ^1^ Department of Clinical Pharmacy, Hainan Cancer Hospital, Haikou, China; ^2^ Department of Hematology, The First Affiliated Hospital of Hainan Medical University, Haikou, China

**Keywords:** cell vesicle, Catharanthus roseus(L.) G. Don, vincristine, drug delivery, cell apoptosis

## Abstract

Vinca alkaloids, a class of naturally derived antimitotic agents isolated from *Catharanthus roseus*, have long been established as potent chemotherapeutic drugs for the treatment of various malignancies. Despite their clinical efficacy, the therapeutic utility of vinca alkaloids, such as vincristine, vinblastine, and vinorelbine, is frequently constrained by systemic toxicity, poor bioavailability, and the emergence of multidrug resistance. In this study, we developed a universal strategy to construct cell membrane-derived vesicles for the encapsulation of vinca alkaloids, thereby enhancing their antitumor efficacy. These artificial cell vesicles were fabricated through the extraction and reconstitution of membranes from K562 cells. Following optimization of drug-loading efficiency, the resulting therapeutic vesicles, designated membrane-encapsulated vinca alkaloids (M@VAs), were thoroughly characterized to evaluate their drug delivery performance. An optimally formulated vincristine-loaded vesicle (M@vincristine) was subsequently used to assess its antitumor efficacy both *in vitro* and *in vivo*. M@vincristine induced a dose-dependent reduction in cell viability and demonstrated significantly greater tumor suppression than free vincristine. At the administered dose, M@vincristine not only promoted enhanced apoptosis but also modulated the expression of key apoptotic factors and effectively induced cell cycle arrest in the M phase. Following intravenous administration, M@vincristine demonstrated efficient tumor accumulation and superior anti-tumor efficacy compared to free vincristine. This artificial therapeutic platform not only addresses the major limitations associated with the clinical use of vinca alkaloids but also paves the way for the development of novel therapeutic agents with broad clinical applicability.

## 1 Introduction

*Catharanthus roseus* (L.) G. Don (Kumar et al., 2022; Sharma et al., 2024; Chaturvedi et al., 2022) is extensively cultivated throughout the southern regions of China, including Guangdong, Guangxi, Yunnan, and Hainan, owing to the favorable tropical and subtropical climates that support its growth and alkaloid production. This medicinal plant is the exclusive natural source of vinca alkaloids, a class of potent naturally derived antimitotic agents that have garnered significant attention in cancer chemotherapy (Chaturvedi et al., 2022; Moudi et al., 2013). Among the most prominent members of this family are vincristine, vinblastine, and their semi-synthetic derivative vinorelbine. These compounds exert their antitumor effects primarily through high-affinity binding to tubulin, a key component of the microtubule cytoskeleton. By inhibiting microtubule polymerization and disrupting mitotic spindle assembly during cell division, vinca alkaloids induce metaphase arrest and ultimately trigger apoptosis in rapidly proliferating tumor cells (Shukla et al., 2024). Due to their potent cytostatic properties, vinca alkaloids have become integral components of clinical oncology, demonstrating broad-spectrum efficacy against hematological malignancies such as leukemias and lymphomas, along with solid tumors including breast cancer, non-small-cell lung cancer, and testicular cancer. Despite their well-established therapeutic value, the clinical utility of vinca alkaloids is substantially constrained by several pharmacological and biological challenges. Systemic toxicity remains a major concern, with dose-limiting adverse effects such as myelosuppression, peripheral neuropathy, gastrointestinal dysfunction, and alopecia, frequently necessitating dose adjustments or treatment discontinuation (Ahmadi et al., 2023; Zhao et al., 2025). Furthermore, their poor bioavailability, which is attributed to low aqueous solubility, extensive hepatic metabolism, and variable absorption, further complicates optimal dosing regimens and limits therapeutic outcomes. These limitations underscore the urgent need for novel strategies, including structural modification, advanced drug delivery systems, and combination therapies, to enhance the therapeutic index and clinical applicability of vinca alkaloids in modern cancer treatment (Ahmadi et al., 2023; Deng et al., 2025; Gu et al., 2021). Nanotechnology has emerged as a transformative approach to address these challenges using engineering drug delivery systems at the nanoscale. Nanocarriers (Farokhzad and Langer, 2009; Mu et al., 2020; Moradi Kashkooli et al., 2020), such as liposomes, polymeric nanoparticles, dendrimers, micelles, and inorganic nanoparticles, offer unique advantages, including improved drug solubility, controlled release kinetics, enhanced permeability and retention (EPR) effect-mediated passive targeting, and the potential for active targeting through surface functionalization with ligands such as antibodies, peptides, or aptamers (Wang et al., 2024; Cheng et al., 2023; Chen et al., 2024). These systems can protect encapsulated drugs from premature degradation, prolong systemic circulation, and facilitate site-specific accumulation, thereby maximizing therapeutic efficacy while minimizing systemic toxicity (Manzari et al., 2021).

Cell membrane-derived drug delivery systems represent an emerging and highly promising class of biomimetic nanocarriers that leverage the natural composition and functionality of cell membranes to enhance the biocompatibility, targeting efficiency, and therapeutic efficacy of encapsulated agents (Tan et al., 2015; Le et al., 2021). Compared with other kinds of nanocarriers, cell-membrane vesicles present distinct advantages over synthetic and other biological drug delivery carriers. Their endogenous origin confers significantly reduced immunogenicity and toxicity risks, enabling safer repeated administration. By cloaking synthetic nanoparticle cores with membranes derived from natural cells, such as red blood cells (RBCs) (Nguyen et al., 2023), platelets (Nguyen et al., 2023), cancer cells (Zeng et al., 2025), immune cells (Yang et al., 2021), or stem cells (Xiao et al., 2024), these hybrid systems inherit the source cells’ surface proteins, antigens, and lipid compositions, thereby enabling immune evasion, prolonged circulation, and specific homing capabilities. This biomimetic strategy effectively mitigates the immunogenicity and rapid clearance commonly associated with conventional synthetic nanocarriers, while also offering the potential for active targeting through receptor–ligand interactions retained from the parent cell membrane. The versatility of cell membrane-coated nanoparticles has been demonstrated across a broad spectrum of biomedical applications, particularly in oncology, where they facilitate tumor-targeted drug delivery, photothermal therapy, and immunomodulation. For instance, RBC membrane-coated nanoparticles exhibit extended systemic circulation due to the presence of CD47 “don’t eat me” signals (Zhang et al., 2024), whereas cancer cell membrane-coated nanoparticles display homotypic targeting, enabling preferential accumulation in primary and metastatic tumor tissues. Moreover, the integration of multiple membrane types into a single platform, such as hybrid RBC–cancer or platelet–RBC membranes, has further expanded the functional repertoire of these systems, allowing for multitargeted delivery and synergistic therapeutic effects (Pang et al., 2023).

**FIGURE 1:**
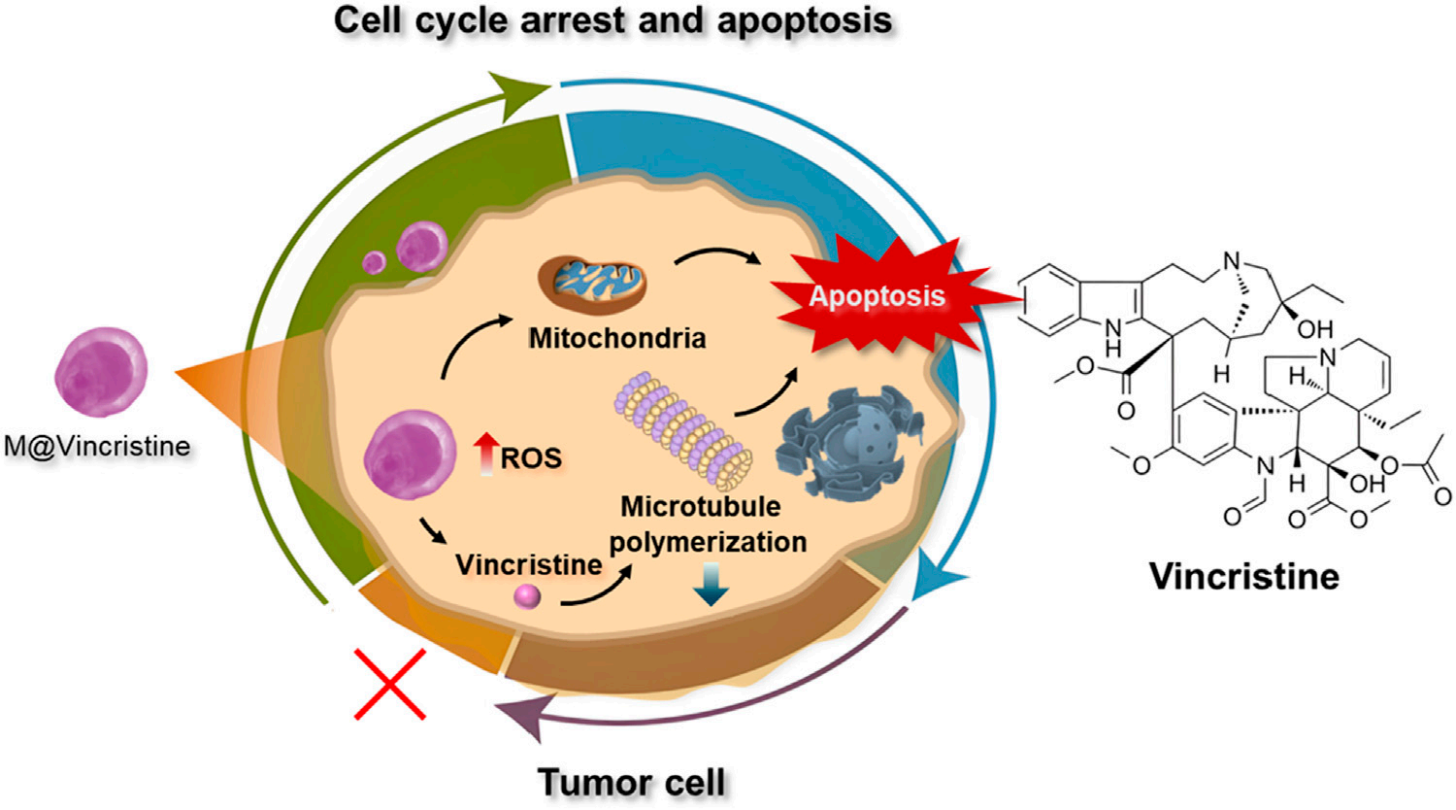
Schematic illustration of the vincristine-loaded artificial cell vesicles and their application in cancer therapy.

In this study, to optimize the therapeutic effect of vinca alkaloids in cancer therapy, we constructed cell membrane-based artificial vesicles designed for the delivery of vinca alkaloids and evaluated their application in cancer treatment (Figure 1). To assess the drug delivery performance, four types of vinca alkaloids, namely, vinblastine, vincristine, vinorelbine, and vindesine, were loaded into the vesicles and characterized in terms of loading efficiency, particle size, and stability. The optimized vincristine-loaded artificial vesicles (M@vincristine) exhibited excellent delivery potential and potent antitumor efficacy in both cellular and murine models. The synergistic effects of enhanced reactive oxygen species (ROS) production, induction of cell apoptosis, and inhibition of microtubule polymerization by M@vincristine collectively contributed to significant tumor cell killing. Our study presents the first example of artificial cell vesicles utilized for vinca alkaloid delivery to enhance cancer therapy.

## 2 Materials and methods

### 2.1 Materials

Vinblastine, vincristine, vinorelbine, vindesine, and fluorescein isothiocyanate (FITC) were purchased from MedChemExpress (MCE, China). Chemicals and solvents were obtained commercially and used without further purification. Millipore water was used to prepare aqueous solutions. DMEM, trypsin, and fetal bovine serum (209111) were purchased from NEST Biotechnology Co. Ltd. (Wuxi, China). The Annexin V-FITC/PI Apoptosis Detection Kit (cat# 40302ES60), Matrigel Matrix LDEV-Free (40183ES10), and Hifair^®^ III One Step RT-qPCR SYBR Green Kit (cat#11143ES70) were purchased from Yeasen, Shanghai, China. Hoechst 33342 and cell count kit-8 (CCK-8) were purchased from Solarbio kit (Beijing, China).

K562 cell was purchased from the American Type Culture Collection. The K562 cells were maintained in DMEM supplemented with 10% FBS and penicillin/streptomycin in a humidified atmosphere containing 5% CO_2_ at 37 °C.

### 2.2 Isolation and purification of the K562 cell membrane

Cell membranes were extracted using a differential centrifugation method, combined with hypotonic treatment and mechanical disruption. In brief, cultured K562 cells were harvested by centrifugation at 500 *g* for 5 min at 4 °C and washed three times with ice-cold phosphate-buffered saline (PBS). The cell pellet was then resuspended in a hypotonic lysis buffer containing 10 mM Tris-HCl, 10 mM KCl, and 1 mM EDTA (pH 7.4) supplemented with protease inhibitors. After incubation on ice for approximately 30 min, the cell suspension was subjected to mechanical disruption using a Dounce homogenizer (50 strokes), followed by sonication on ice (three cycles of 10 s pulse at 30% amplitude with 20 s cooling intervals). The cell lysate was centrifuged at 1,000 × g for 10 min at 4 °C to remove the unbroken cells and nuclei. The resulting supernatant was further centrifuged at 20,000 × g for 30 min at 4 °C to pellet the crude membrane fraction. The membrane pellet was washed twice with PBS and resuspended in an appropriate buffer for subsequent characterization or experimentation. The protein concentration of the membrane fraction was quantified using the BCA protein assay kit.

### 2.3 Construction of the vinca alkaloid-loaded artificial cell vesicles

To construct the vinca alkaloid-loaded artificial cell vesicles, the freshly prepared K562 cell membranes were resuspended in cold PBS, added with vinca alkaloids (vinblastine, vincristine, vinorelbine, or vindesine), and cultured at room temperature for 6 h. The drug concentration ranged from 1 to 5 mg/mL. Then, the mixture was subjected to extrusion through a polycarbonate membrane using a mini-extruder to form the drug-loaded artificial cell vesicles (M@VAs). Unencapsulated vinca alkaloid and free membrane components were removed by centrifugation at 12,000 g for 20 min at 4 °C.

The final product was characterized for measuring the size distribution using dynamic light scattering (DLS). The drug encapsulation efficiency was measured using high-performance liquid chromatography (HPLC). The analysis was performed using a Diamonsil C18 column. The mobile phase consisted of acetonitrile–methanol–diethylamine (13:53:34, V/V/V). The detection wavelength was set at 281 nm, with a flow rate of 1.0 mL/min. The column temperature was maintained at 30 °C, and the injection volume was 10 μL. The drug-loading efficacy (DLE) was calculated according to the following equation:

$$\%\text{ DLE}=\left[\left(\text{Drug added}-\text{Unloaded drug}\right)/\text{ Drug added}\right]\times 100$$

### 2.4 Stability assay

The vinca alkaloid-loaded artificial cell vesicles were dispersed into PBS and incubated under continuous agitation at 37 °C. At predetermined time points (0 h, 3 h, 6 h, 12 h, 24 h, 48 h, and 72 h), a 1-mL aliquot of the culture was withdrawn and characterized for size distribution using DLS.

### 2.5 Cellular cytotoxicity assay

Cellular viability was evaluated using the CCK-8 assay. In brief, K562 cells were seeded into 96-well plates at a density of 5,000 cells per well and allowed to adhere overnight in a humidified incubator at 37 °C under 5% CO_2_. Following adhesion, the culture medium was replaced with fresh medium supplemented with varying concentrations of the test agents, including PBS, cell membrane, free vincristine, or M@vincristine. After a 72-h incubation period, the medium was carefully aspirated, and 100 µL of fresh culture medium containing 10% (v/v) CCK-8 reagent was added to each well. The plates were then incubated for an additional 1 h at 37 °C. Finally, the absorbance of each well was measured at 450 nm using a microplate reader, with a reference wavelength of 630 nm to correct for nonspecific background absorbance.

### 2.6 Animals and tumor model establishment

For the animal test, female BALB/c mice, aged 4–6 weeks, were procured from Beijing Vital River Laboratory Animal Technology Co., Ltd. All experimental procedures involving animals were conducted in accordance with the guidelines approved by the Institutional Animal Care and Use Committee of Hainan Medical University (HYLL-2023-379). To establish the mouse tumor model, 5 × 10^5^ K562 cells were suspended in 50 μL of Matrigel and subcutaneously inoculated into the mice. The experiment was initiated once the tumor volume reached approximately 100 mm^3^. Each experimental group consisted of n = 5 mice.

### 2.7 Statistical analysis

In this study, all data are presented as the mean ± standard deviation (s.d.) from the indicated number of independent replicates. Statistical analyses were performed using GraphPad Prism software. For comparisons between two groups, statistical significance was determined using the unpaired two-tailed Student’s t-test. A P-value of less than 0.05 was considered to indicate statistical significance.

## 3 Results and discussion

### 3.1 Construction of the vinca alkaloid-loaded artificial cell vesicles

**FIGURE 2:**
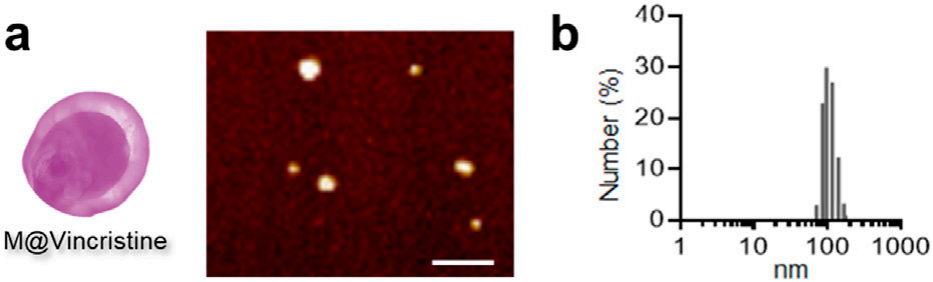
Characterization of M@vincristine. **(a)** AFM images of M@vincristine. Scale bar: 500 nm. **(b)** Hydrodynamic size analysis of M@vincristine measured through DLS analysis.

Based on our previous research experience, we utilized the differential centrifugation method, combined with hypotonic treatment and mechanical disruption, to prepare the extracted cell membrane. We constructed the vinca alkaloid-loaded artificial cell vesicles using K562 cell membranes and aimed to apply them in the treatment of chronic myelogenous leukemia. In our study, four types of vinca alkaloids (vinblastine, vincristine, vinorelbine, and vindesine) were loaded in the artificial cell vesicles through non-covalent on covalent encapsulation. The DLE of the purified M@VA was measured using HPLC. A relatively high vinca alkaloid loading was obtained through the universal and convenient loading strategy (vinblastine: 116.8 μg/mg vesicle, vincristine: 176.5 μg/mg vesicle, vinorelbine: 109.4 μg/mg vesicle, and vindesine: 88.4 μg/mg vesicle). The corresponding DLE analysis indicated that the M@vincristine exhibited the best loading effect (vinblastine: 23.4% ± 2.2%, vincristine: 35.3% ± 3.1%, vinorelbine: 21.9% ± 4.0%, and vindesine: 17.7% ± 3.8%). Next, we tested the particle size of the four types of M@VA. All of the four types of M@VA were of a uniform size of approximately 100 nm, according to the results of the DLS in PBS. Nevertheless, the polydispersity index (M@vincristine: 0.195) reveals greater uniformity in M@vincristine, making it more appropriate for use as drug delivery vehicles. M@vincristine was further characterized with uniform morphology according to the AFM images, and the diameter of it was 109.7 ± 10.4 nm (Figures 2a,b). In the stability assay, M@vincristine remained stable in the physiological environment for 72 h, indicating excellent drug delivery performance. Overall, M@vincristine would be favorable for application in cancer treatment.

### 3.2 Synergistic tumor inhibition

**FIGURE 3:**
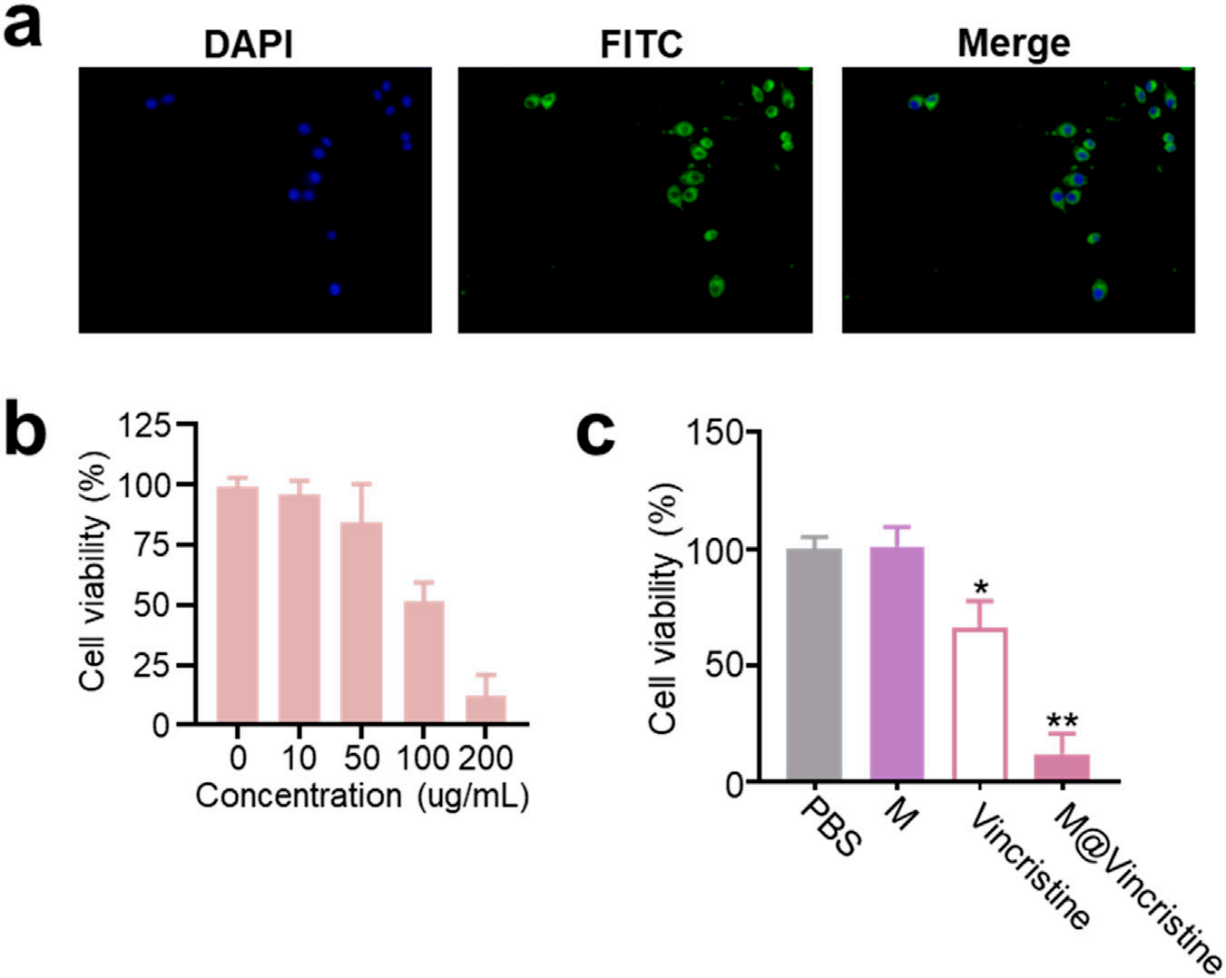
Cellular drug delivery performance of M@vincristine. **(a)** Confocal images of K562 cells cultured with FITC-labeled M@vincristine for 3 h at 37 °C. For fluorescence labeling, M@vincristine was cultured with FITC (1 mM) in PBS for 3 h at room temperature, and extra FITC was removed through ultrafiltration. **(b)** Relative cell viability of K562 cells after the treatment of different concentrations of M@vincristine. The drug concentration ranged from 0 to 200 ug/mL. **(c)** Relative cell viability of K562 cells after different treatment. Data are expressed as mean ± SEM of three independent experiments. (*p < 0.05 and **p < 0.01).

We then proceeded to evaluate the drug delivery effects of the artificial cell vesicles at the cellular level on K562 cells. To verify the internalization of M@vincristine, fluorescence probe FITC was introduced for confocal image analysis. As shown in Figure 3a, a significantly clear fluorescence signal was detected in the FITC-labeled M@vincristine treatment group, indicating effective internalization of the vesicle. To evaluate the potential inhibition effect, we first investigated the cytotoxicity of M@vincristine using the CCK8 assay, as shown in Figure 3b. After 72 h of incubation, M@vincristine showed dose-dependent cytotoxicity at a relatively low concentration. M@vincristine exhibited a significantly higher cell inhibition effect than free vincristine at the same dose, indicating excellent therapeutic potential (Figure 3c).

**FIGURE 4:**
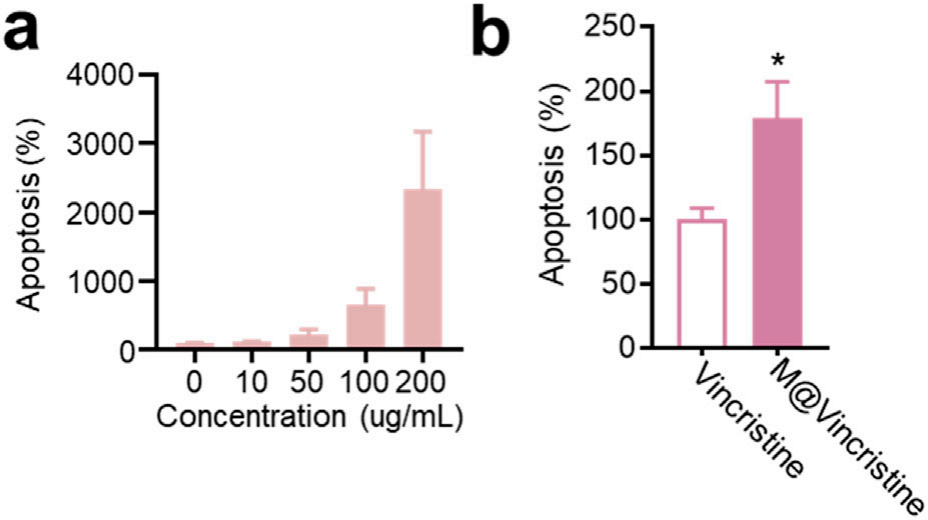
Dose-dependent apoptosis effect induced by M@vincristine in K562 cells. **(a)** Relative apoptosis of K562 cell after the treatment of different concentrations of M@vincristine from 0 to 200 ug/mL. **(b)** Cell apoptosis analysis of cells after the treatment of PBS, free vincristine, or M@vincristine. The drug concentration was based on 200 μg/mL of M@vincristine. Data are expressed as mean ± SEM of three independent experiments. (*p < 0.05 and **p < 0.01).

In the apoptosis assay conducted using the Annexin V-FITC/PI Apoptosis Detection Kit, M@vincristine also showed dose-dependent apoptosis induction in K562 cells, as shown in Figure 4a. As expected, a remarkably enhanced effect in cell inhibition was achieved by M@vincristine compared with free vincristine (Figure 4b).

### 3.3 Mechanism analysis

**FIGURE 5:**
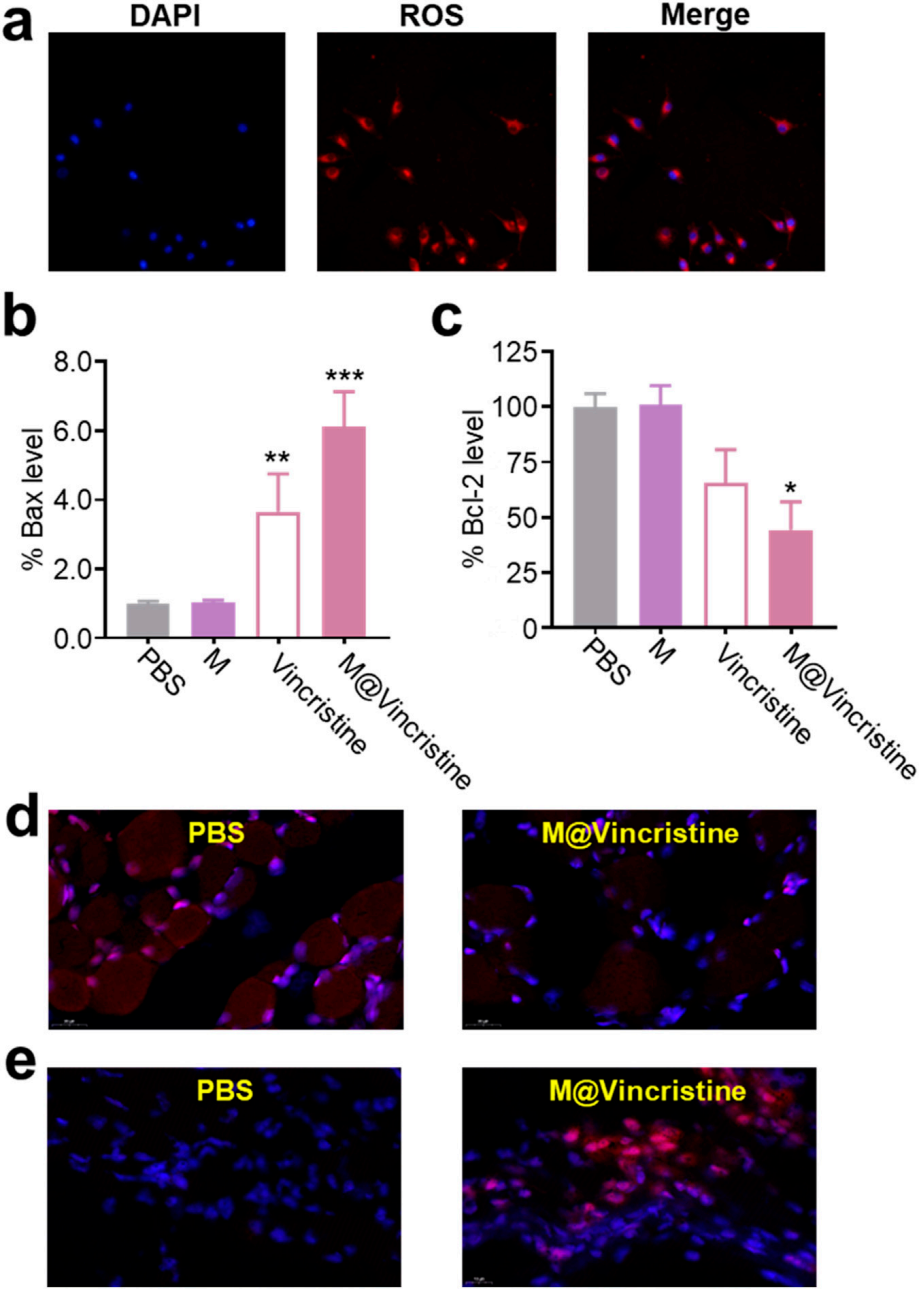
Mechanism analysis of M@vincristine in apoptosis induction. **(a)** ROS imaging of K562 cells treated with M@vincristine. **(b)** Relative Bax level of K562 cells measured through qPCR. **(c)** Relative Bcl-2 level of K562 cells measured through qPCR. **(d)** Immunofluorescence staining of Bcl-2. **(e)** Immunofluorescence staining of Bax. Data are expressed as mean ± SEM of three independent experiments. (*p < 0.05, **p < 0.01, and ***p < 0.001).

In the mechanism analysis, we tested molecular markers of apoptosis (Bax and Bcl-2) and microtubule polymerization, as shown in Figure 1. Accumulating evidence suggests that a sustained increase in intracellular ROS levels is closely associated with the induction of apoptosis, a form of programmed cell death essential for maintaining tissue homeostasis and eliminating damaged cells. ROS-mediated apoptosis is primarily triggered through the intrinsic (mitochondrial) pathway, where elevated ROS promotes mitochondrial membrane permeabilization, cytochrome C release, and activation of caspase cascades (including Bax and Bcl-2) (Simon et al., 2000; Holze et al., 2018). As shown in Figure 5a, the treatment of the M@vincristine (200 μg/mL) in 24 h notably induced fluorescence signals of ROS in K562 cells. Meanwhile, the level of the apoptosis-related Bax and Bcl-2 remarkably regulated under the treatment of M@vincristine, indicating effective regulation in induing cell apoptosis (Figures 5b–e).

**FIGURE 6:**
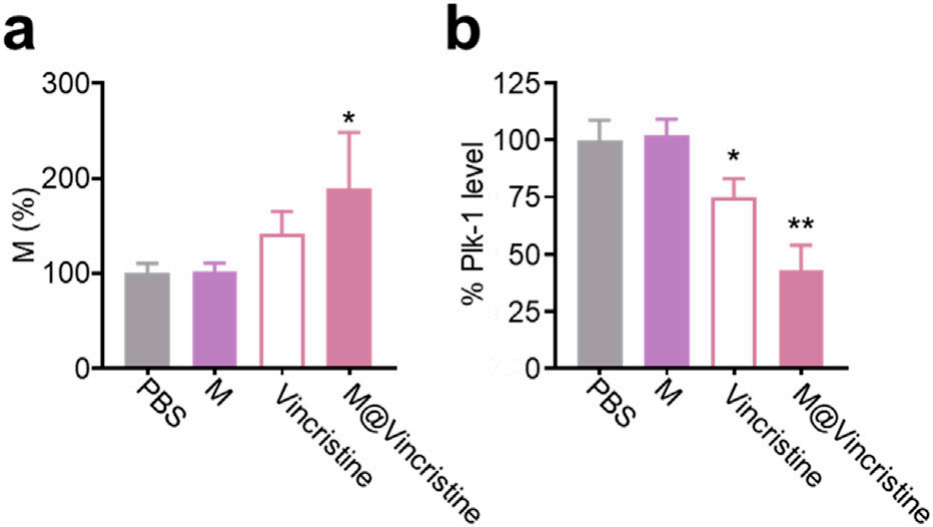
Mechanism analysis of M@vincristine in microtubule polymerization inhibition. **(a)** Cell cycle assay of K562 cells under the treatment of PBS, M, vincristine, or M@vincristine. The treated cells were stained with PI and analyzed through flow cytometry. **(b)** Relative Plk-1 level of K562 cells measured through qPCR. Data are expressed as mean ± SEM of three independent experiments. (*p < 0.05 and **p < 0.01).

Next, we investigated the therapeutic mechanism of M@vincristine, focusing on the effects on microtubule polymerization. Microtubules, which are the key components of the cytoskeleton, play a critical role in maintaining cell structure, enabling intracellular transport, and facilitating cell division. During mitosis, the proper assembly and disassembly of microtubules are essential for spindle formation, chromosome polymerization, and cytokinesis (Choudhury et al., 2013). Dysregulation of microtubule dynamics, such as aberrant microtubule polymerization, can disrupt these processes, leading to cell cycle arrest and impaired cell proliferation (Choudhury et al., 2013; Fan et al., 2020). Polo-like kinase 1 (PLK1), a serine/threonine kinase, is a crucial regulator of mitotic progression. It is involved in multiple mitotic events, including centrosome maturation, spindle assembly, and chromosome segregation. The expression and activity of PLK1 are tightly controlled during the cell cycle, and its dysregulation has been implicated in various malignancies (Hugle et al., 2015; Ebisu et al., 2021; Xu and Dai, 2011). According to the results of the cell cycle assay, following treatment with M@vincristine, the proportion of cells in the M phase of the cell cycle was significantly increased, particularly when compared to the group treated with free vincristine under the indicated doses (Figure 6a). Correspondingly, the Plk-1 level sharply decreased in the M@vincristine group (Figure 6b).

### 3.4 *In vivo* anticancer therapy

**FIGURE 7:**
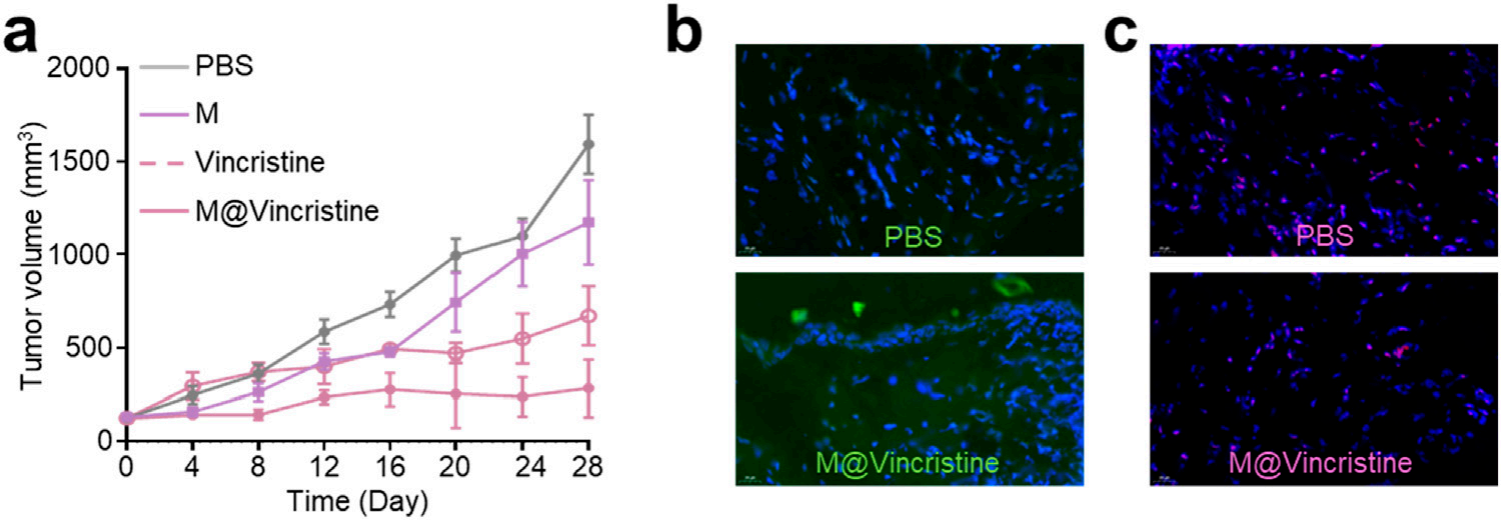
**(a)** Tumor volume curve of each treatment group. **(b)** Images of immunofluorescence staining of Bax. Nucleus was stained with DAPI. **(c)** Images of immunofluorescence staining of PLK-1. Nucleus was stained with DAPI.

Encouraged by the excellent *in vitro* therapeutic performances of M@vincristine, we next proceeded to study the *in vivo* anticancer efficacy. The *in vivo* tumor suppression efficiency was assessed in a K562 tumor model. PBS, M, GE@Cur, and ^A^GE@Cur (based on 2 mg vincristine/kg body weight) were administered via tail vein injection. The administration was conducted on days 0, 4, and 8. Tumors in the PBS control demonstrated a precipitous growth trajectory over the treatment. In contrast, treatment with M@vincristine significantly impeded the tumor proliferation, which aligns with the *in vitro* cytotoxicity assessments, as expected (Figure 7a). Next, we conducted an immunofluorescence analysis of the Bax and PLK-1 protein levels within the tumor tissue following various treatments. As shown in Figure 7b, a marked increase in the pro-apoptotic factor Bax was measured. Meanwhile, a significant reduction in PLK-1 levels was evident following M@vincristine treatment (Figure 7c), indicating effective regulation of the tumor apoptosis. In addition, the body weights of mice during the treatment were similar and steadily increased, providing strong evidence for the biosafety of the artificial cell vesicles (Supplementary Figure S2). The above results suggested that M@vincristine holds exceptional therapeutic effects in cancer treatment and opens an avenue for the development of the vinca alkaloid-loaded artificial cell vesicles.

## 4 Conclusion

In conclusion, this study successfully developed a novel cell membrane-derived vesicle-based delivery system for vinca alkaloids, designated M@VA, which effectively overcomes the major limitations associated with their clinical application, including systemic toxicity, poor bioavailability, and multidrug resistance. Through the optimized encapsulation (17%–35%) of vincristine into artificial vesicles derived from K562 cell membranes, the resulting M@vincristine formulation demonstrated superior antitumor efficacy both *in vitro* and *in vivo*. Notably, M@vincristine exhibited enhanced tumor suppression, dose-dependent cytotoxicity, and efficient tumor-targeting delivery following intravenous administration. Mechanistically, M@vincristine not only induced significant apoptosis but also modulated the expression of key apoptotic factors and triggered M-phase cell cycle arrest, highlighting its potent antitumor activity. These findings underscore the potential of M@VA as a versatile and effective platform for the delivery of chemotherapeutic agents, offering a promising strategy to enhance the therapeutic index of vinca alkaloids and paving the way for the development of next-generation, clinically translatable nanomedicines.

## Data Availability

The original contributions presented in the study are included in the article/Supplementary Material; further inquiries can be directed to the corresponding authors.
