# Supplementary material for: Artificial cell vesicle-mediated delivery of Catharanthus roseus (L.) G. Don-derived vinca alkaloids for enhanced antitumor efficacy
Source: Front Bioeng Biotechnol. 2025 Oct 15;13:1703637. doi: 10.3389/fbioe.2025.1703637 (PMC12568567; doi:10.3389/fbioe.2025.1703637)
Supplement: Supplementary file 1 [file Supplementaryfile1.docx]

Supplementary Material

Artificial Cell Vesicle-Mediated Delivery of *Catharanthus roseus* (L.) G. Don-derived Vinca Alkaloids for Enhanced Antitumor Efficacy

Xiaodong Zhuang^1^, Liangjiu Huang ^1^, Risheng Liu^1^, Liwen Guan^1^, Xingyue Fang^2*^, Ting Ma ^1*^

1 Department of Clinical Pharmacy, Hainan Cancer Hospital, Haikou, 570312, China

2 Department of Hematology, The First Affiliated Hospital of Hainan Medical University, Haikou, 570102, China

.

*** Correspondence:**Ting Ma

hyfzl00555@muhn.edu.cn

# Supplementary Figures


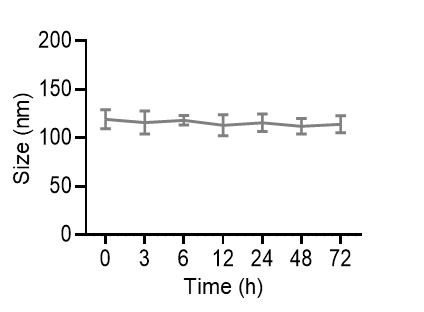


**Supplementary Figure 1.** DLS analysis of the M@vincristine.


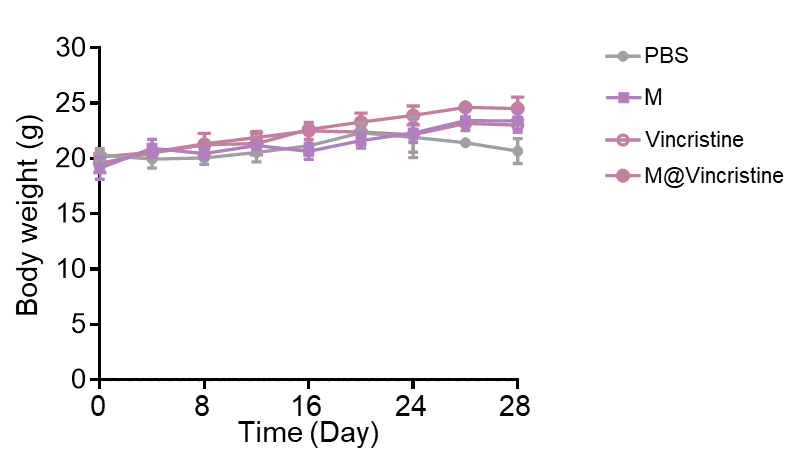


**Supplementary Figure 2.** Body weight analysis of the mice under different treatment.
